# Roles of Phase Separation for Cellular Redox Maintenance

**DOI:** 10.3389/fgene.2021.691946

**Published:** 2021-07-09

**Authors:** Yuichi Saito, Wataru Kimura

**Affiliations:** Laboratory for Heart Regeneration, RIKEN Center for Biosystems Dynamics Research, Kobe, Japan

**Keywords:** liquid-liquid phase separation, redox biology, oxidative stress, autophagy, Nrf2, hypoxia

## Abstract

The oxidation reaction greatly alters characteristics of various cellular components. In exchange for efficient energy production, mitochondrial aerobic respiration substantially increases the risk of excess oxidation of cellular biomolecules such as lipids, proteins, nucleic acids, and numerous small molecules. To maintain a physiologically balanced cellular reduction-oxidation (redox) state, cells utilize a variety of molecular machineries including cellular antioxidants and protein degradation complexes such as the ubiquitin-proteasome system or autophagy. In the past decade, biomolecular liquid-liquid phase separation (LLPS) has emerged as a subject of great interest in the biomedical field, as it plays versatile roles in the maintenance of cellular homeostasis. With regard to redox homeostasis, LLPS arose as a major player in both well-characterized and newly emerging redox pathways. LLPS is involved in direct redox imbalance sensing, signal transduction, and transcriptional regulation. Also, LLPS is at play when cells resist redox imbalance through metabolic switching, translational remodeling, activating the DNA damage response, and segregation of vulnerable lipids and proteins. On the other hand, chronic accumulation of phase-separated molecular condensates such as lipid droplets and amyloid causes neurotoxic outcomes. In this review we enumerate recent progress on understanding how cells utilize LLPS to deal with oxidative stress, especially related to cell survival or pathogenesis, and we discuss future research directions for understanding biological phase separation in cellular redox regulation.

## Introduction

Oxygen is a major electrophilic element which can easily produce excessively electrophilic molecules called reactive oxygen species (ROS) (Auten and Davis, 2009). Organisms utilize the high electrophilicity of oxygens as a driving force for various cellular chemical reactions. For example, mitochondria use oxygen molecules as an electron acceptor for efficient ATP production via the electron transport chain, at the expense of generating ROS as a byproduct (Zorov et al., 2014).

**FIGURE 1:**
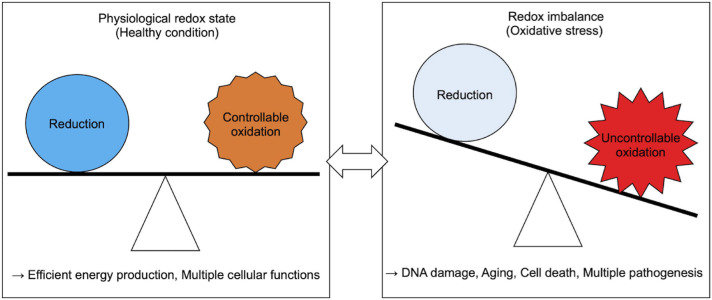
Physiological and pathological cellular redox state.

Generally, acute and chronic redox imbalance in cells results in oxidative stress (Sies, 2015). Free ROS oxidize various intracellular organelles, macromolecules, and small molecules, and impair their function (Figure 1). For example, the purine nucleotide guanine is susceptible to oxidation, which leads to DNA mutations that may initiate and propagate some types of cancers (Lonkar and Dedon, 2011; Mangerich et al., 2012; Cadet and Davies, 2017). Peroxidized lipids, especially polyunsaturated fatty acids (PUFAs), impair cellular membrane integrity and propagate further production of ROS (Farmer et al., 1943; Gardner, 1989; Gaschler and Stockwell, 2017), and activate oxidative cell death signaling (Gaschler and Stockwell, 2017). Furthermore, proteins are also major targets of peroxidation. Proteins containing electron-rich amino acid residues are susceptible to oxidation, which is associated with the progression of age-related phenotypes observed in premature aging disorders (Stadtman, 1992, 2001). To prevent these pathogenic outcomes induced by redox imbalance, cells need to maintain cellular redox homeostasis.

Because free ROS quickly induce this oxidation-mediated cellular damage, excess oxidation needs to be dealt with through oxidative stress response systems. For example, thiol oxidation in Keap1 (Kelch-like ECH-associated protein 1) triggers the activation of antioxidant response protein Nrf2 [nuclear factor erythroid 2 (NF-E2)-related factor 2] (Suzuki and Yamamoto, 2015). In contrast, how redox imbalance changes the behavior of a large number of cellular biomolecules to activate redox response pathways or that triggers pathological events has been largely unknown.

Brangwynne et al. (2009) reported liquid-like dynamic properties of the cytoplasmic structures known as germline P granules in *C. elegans* embryos. This study showed that P granules are dissolved and condensed, enabling a rapid exchange of their components (Brangwynne et al., 2009). This phase structuring is now considered to be a general mechanism for assembly of biomolecules, especially macromolecules such as proteins, nucleic acids and lipids in cells. In recent years biomolecular liquid-liquid phase separation (LLPS) has gained a lot of interest in understanding the molecular rules that determine multiple biological events, such as transcriptional regulation (Sabari et al., 2018), signal transduction (Su et al., 2016; Banani et al., 2017), and protection of macromolecules from toxic conformation changes (Riback et al., 2017). Not surprisingly, LLPS mediates biological events that are associated with redox maintenance by modifying phase behavior of macromolecules (Table 1). Physical properties of phase-separated liquid droplets provide several critical aspects of redox maintenance reactions, such as rapid and reversible sensing of and response to the redox imbalance. In this review, we focus on how cells utilize LLPS for redox maintenance *in vivo* and summarize current research on phase separation-mediated cellular redox sensing and response. We also discuss future research directions for better understanding of the etiology of and advancing therapeutic approaches for redox-related diseases.

**TABLE 1:** Roles of phase separation-associated biological events related to redox maintenance.

**LLPS-mediated redox imbalance sensing**		
**Phase separation events**	**Components**	**Biological roles**
LC domain self-association	Pbp1	Mediates redox reactive phase separation, Activates autophagy
Signal transduction	MAPK related proteins	Quickly transduces MAPK signals
p62 droplet	p62	Is targeted for autophagic degradation, Activates Nrf2
Super enhancer	Transcription factors	Efficiently activates transcription

**LLPS-mediated resistance for redox imbalance**		

**Phase separation events**	**Components**	**Biological roles**
Glycolytic body (G body)	Glycolytic enzymes	Efficiently produce energy by glycolysis
Stress granule (SG)	RNA, mTOR, etc.	Protects untranslated RNAs from oxidative damage
P body	RNA, etc.	Protects untranslated RNAs from oxidative damage
53BP1 droplet	53BP1, p53	Induces p53 and p53 target genes
Lipid droplet	Polyunsaturated fatty acids (PUFAs)	Protects PUFA from lipid peroxidation, Causes neurotoxicity when accumulated
Amyloid formation	α-Synuclein and Amyloid β, etc	Protects proteins from oxidative damage, Causes neurotoxicity when accumulated

## Cellular Redox State

### Physiological Usage of Redox Reactions

Diverse redox reaction-related enzymes produce ROS (Sies and Jones, 2020). NADPH oxidase (NOX) family enzymes transport electrons across the plasma membrane and generate O_2_^–^ or H_2_O_2_ (Bedard and Krause, 2007). NOX enzymes contribute to various physiological events, such as host defense, cellular signaling, and cell differentiation (Leto and Geiszt, 2006; Bedard and Krause, 2007; Hahner et al., 2020). In the host defense system, generated ROS are used for deletion of the harmful exogenous substances. For example, phagocytes such as macrophages generate ROS through phagosomal NOX machinery and play a role in killing bacteria (Lambeth, 2004).

Peroxisomes contain several types of oxidases and catalase (Schrader and Fahimi, 2006; Fransen et al., 2012). Using these redox enzymes, peroxisomes are involved in various aspects of cellular metabolism including long fatty acid oxidation, purine catabolism, and polyamine catabolism (Lazarow, 1987).

Mitochondria also generate ROS as a consequence of aerobic energy production (Zorov et al., 2014). Mitochondrial ROS transduce both physiological and pathological cellular signaling (Starkov, 2008; Lenaz, 2012; Sena and Chandel, 2012; Angelova and Abramov, 2016). Similar to ROS produced by phagocytes, mitochondrial ROS contribute to the bactericidal activity in macrophages via TLR signaling (West et al., 2011). Furthermore, mitochondrial ROS regulate the cell cycle in a variety of cellular contexts. For example, cardiomyocyte cell cycle arrest in the postnatal mammalian heart is mediated by mitochondrial ROS as a result of metabolic adaptation to postnatal life. In mice, the postnatal transition from a hypoxic intrauterine environment to an oxygen-rich postnatal environment increases mitochondrial respiration (Puente et al., 2014). Increased mitochondrial activity leads to the elevation of mitochondrial ROS production, which causes cell cycle arrest in mammalian cardiomyocytes (Puente et al., 2014). On the other hand, pathological insults by mitochondrial ROS are triggered by metabolic dysregulation or exposure to excess oxidants which leads to redox imbalance, as described in the following section.

### Cause of Cellular Redox Imbalance

Redox imbalance by an excess of ROS occurs for a variety of reasons. Firstly, various chemical substances (e.g., dietary compounds, heavy metals, and pharmaceutical metabolites) called xenobiotics cause ROS production (Klotz and Steinbrenner, 2017). Experimentally, arsenites are often used as an inducer of ROS production both *in vitro* and *in vivo* (Ruiz-Ramos et al., 2009; Flora, 2011). Secondly, damaged organelles also often lead to outcomes that are detrimental to cells, mainly by the leakage of their components which sometimes include the sources of ROS production. For example, lysosomes contain various types of proteases (e.g., cathepsins) and ROS itself, and therefore, lysosomal rupture or membrane permeabilization causes oxidative insults (Zhao et al., 2003; Ghosh et al., 2011). Mitochondrial dysfunction also causes oxidative insults. As we will discuss later in this review, recent studies have shown that reverse electron transfer in mitochondrial complex I serves as an important source of pathological ROS production (Murphy, 2009; Robb et al., 2018; Yin et al., 2021). A third cause of redox imbalance is the reduction of antioxidant levels, for example by the down-regulation of antioxidant capacities during the aging process (Zhang et al., 2015; Kubben et al., 2016).

Acute environmental changes can cause oxidative insults. For example, acute hypoxia induces ROS production via metabolic perturbation (McGarry et al., 2018). In addition, ischemia-reperfusion also causes ROS production, which contributes to pathology of myocardial and cerebral infarction (Cadenas, 2018; Wu et al., 2020). In ischemia-reperfusion pathology, reoxygenation by reperfusion accelerates ROS production by enzymes such as Xanthine oxidase or NADPH oxidases, and from mitochondria at least in part through reverse electron transfer at mitochondrial complex I caused by accumulated succinate during ischemia (Chouchani et al., 2014; Granger and Kvietys, 2015), as we will discuss later.

Thermal stress induced by heat or cold also causes redox imbalance due to overproduction of mitochondrial or other sources of ROS (Ali et al., 2010; Slimen et al., 2014; Sun et al., 2016; Ou et al., 2018). Heat stress impairs mitochondrial ATP synthesis and increases superoxide anion production (Slimen et al., 2014). Cold stress also induces ROS generation through ion channels (Sun et al., 2016) and lysosomal membrane permeabilization, both of which lead to tubulin damage (Ou et al., 2018). Interestingly, a moderately lower temperature leads to a reduction in mitochondrial respiration rate, which unexpectedly also causes an increase in ROS production (Ali et al., 2010). Thus, maintenance of proper body temperature is critical for the regulation of cellular redox balance.

Solar irradiation, especially ultraviolet (UV) and short-wavelength visible light exposure, produces ROS at the body surface and retina (Kuse et al., 2014; de Jager et al., 2017; Nakashima et al., 2017). As shorter wavelength radiation has greater energy, light-induced ROS production mainly depends on energy levels. In addition to an exposure to UV-induced ROS, DNA directly absorbs UV, and receives damages (Markovitsi et al., 2010). On the contrary, weak long-wavelength light exposure decreases ROS, a process called photobiomodulation (Hamblin, 2018). The proposed mechanism of photobiomodulation also relates to selective light absorption by biomolecules. For example, red and near-infrared light is absorbed by mitochondrial cytochrome c oxidase and causes photodissociation of NO, which inhibits mitochondrial respiration and ATP production by cytochrome c oxidase (Sarti et al., 2012; Hamblin, 2018).

In addition to these environmental stresses, specific cellular status such as inflammation or starvation causes ROS production (Liu et al., 2003; Mittal et al., 2014). Inflammation, which is induced by infection or tissue injury, shares many features with the oxidative stress condition (Medzhitov, 2008; Mittal et al., 2014). Starvation, especially glucose deprivation which is used for *in vitro* model of ischemic stroke in combination with oxygen deprivation (Tasca et al., 2015), causes excess ROS production and ATP depletion due to lack of glucose as the mitochondrial energy source, and the starvation-induced ROS triggers starvation-induced autophagy (Liu et al., 2003; Scherz-Shouval et al., 2007; Filomeni et al., 2015). Taken together, redox imbalance occurs in diverse conditions which in most cases is associated with the disturbance of mitochondrial metabolism.

### Maintenance of Redox Homeostasis Through Redox Imbalance Responses

**FIGURE 2:**
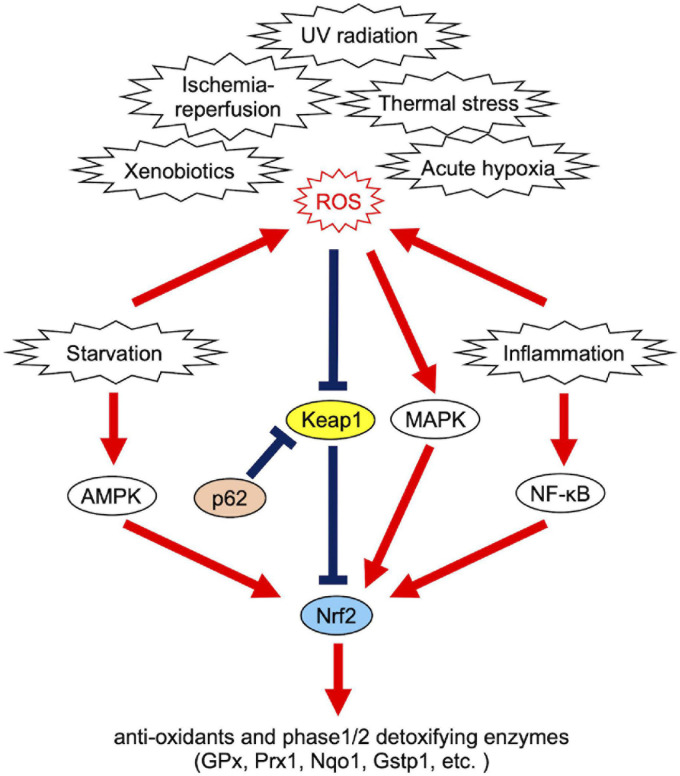
Causes of redox imbalance and Nrf2-associated cellular response systems.

Reactive oxygen species production is not always harmful to organisms. Physiologically controllable ROS increase stress resistance and extend lifespan through activation of the stress response systems (Sies and Jones, 2020). This effect is called oxidative eustress or hormesis. However, uncontrollable ROS, namely redox imbalance, are detrimental to normal cellular functions. To deal with excessively produced ROS in diverse conditions, cells have various stress response systems including the production of antioxidant enzymes such as glutathione peroxidase (GPx) or peroxiredoxin 1 (Prx1) (Deponte, 2013; Ledgerwood et al., 2017). Nrf2 [nuclear factor erythroid 2 (NF-E2)-related factor 2], a master regulator transcription factor of cellular redox maintenance, regulates expression levels of these antioxidant enzymes to reduce ROS, and phase1/2 detoxifying enzymes to remove ROS-generating xenobiotics [e.g., NADPH quinone dehydrogenase 1 (Nqo1) and glutathione S-transferase class Pi 1 (Gstp1)] (Deponte, 2013; Hayes and Dinkova-Kostova, 2014). Phase 1 enzymes catalyze oxidation, reduction and hydrolysis reactions and phase 2 enzymes catalyze conjugation reactions (Iyanagi, 2007). Nrf2 is activated as a result of crosstalk with several stress response signals including NF-κB (nuclear factor kappa-light-chain-enhancer of activated B cells) or AMPK (5’ adenosine monophosphate-activated protein kinase) (Mo et al., 2014; Wardyn et al., 2015; Joo et al., 2016; Sivandzade et al., 2019), and thus Nrf2 acts as an oxidative stress signaling hub (Figure 2). These regulatory cascades are required to manage the production of secondary ROS in diverse stress conditions, such that inflammation activates NF-κB signaling, and starvation activates AMPK signaling (Morgan and Liu, 2011; Ren and Shen, 2019). Also, mitogen-activated protein kinase (MAPK) signaling quickly transmits oxidative stress information and activates stress responsive transcription factors, including Nrf2 (Shen et al., 2004; Keum et al., 2006). Other than Nrf2, FOXO (forkhead box, class O) transcription factors and PPARs (peroxisome proliferator–activated receptors) are also known as antioxidant regulators (Devchand et al., 2004; Klotz et al., 2015). Recently, a series of discoveries related to biological phase separation has given the field of redox biology additional significance. Cellular redox maintenance utilizes phase separation in a variety of ways for sequestration of vulnerable cellular components, as discussed in the following sections.

## LLPS in Redox Imbalance Sensing

### Sensing Redox States

Protein low complexity (LC) domains have been found in various proteins including RNA or DNA binding proteins as essential domains for transcription activation, mRNA processing, or nuclear transport (Dyson and Wright, 2005). Recent advances in the field of LLPS biology have shown the importance of the LC domain to undergo LLPS (Banani et al., 2017). In addition, post-translational modification of the LC domains directly senses redox imbalance and changes phasing properties of proteins. Kato et al. (2019, 2021) found that Pbp1 (poly-A binding protein binding protein) directly responds to redox imbalance. Pbp1 is a yeast ortholog of ataxin 2, a neurodegenerative disease-associated protein (Lastres-Becker et al., 2008; Da Conceição Alves-Cruzeiro et al., 2016). The methionine-rich LC domain of Pbp1 is prone to be oxidized upon oxidative stress induced by dysfunctional mitochondria or H_2_O_2_ (Kato et al., 2019, 2021). Oxidized methionines in the LC domain inhibit self-association of LC domains in Pbp1, resulting in dissolution of Pbp1 droplets to activate target of rapamycin complex 1 (TORC1) and TORC1-mediated autophagy (Kato et al., 2019, 2021). Since autophagy works for clearance of damaged organelles and stress granules (SGs) (Buchan et al., 2013; Anding and Baehrecke, 2017), ROS-induced melting of Pbp1 droplets may be necessary for clearance of oxidatively damaged cellular components. The direct reaction of the LC domain against oxidants is also observed in TDP43 (transactivation response DNA-binding protein of 43 kDa), indicating a shared mechanism for direct redox sensing by LC domains to regulate association or dissociation of the LC domain itself to activate cellular oxidative stress response pathways (Kato et al., 2021).

### Signal Transduction

When redox imbalance exceeds a certain threshold, cells initiate signal transduction to handle oxidative stress. MAPK cascades activate Nrf2-mediated oxidative stress responses (Figure 2). Banani et al. (2017) proposed that phase-separated liquid compartments can give a specificity to signaling cascades that potentially activate multiple biological processes by incorporating reaction substrates of one specific pathway and excluding others. Su et al. (2016) suggested that this is the case with the MAPK signaling pathway; phase separation of LAT (linker for activation of T cells) promotes signaling including MAPK (ERK) in T cells. In this context, LAT clustering concentrates ZAP70 which phosphorylates LAT, and excludes CD45 which dephosphorylates p-LAT (Su et al., 2016). In addition to pathway choice, LLPS is also involved in disease-associated MAPK hyperactivation during development. Zhu et al., showed that developmental disease-associated mutations in SHP2, the non-receptor protein tyrosine phosphatase, facilitate the formation of liquid droplets that promote MAPK activation (Zhu et al., 2020). Importantly, SHP2 allosteric inhibitors block LLPS of mutant SHP2, and thus LLPS can be therapeutically targeted for the treatment of developmental diseases (Zhu et al., 2020). Taken together, LLPS plays critical roles in the regulation of activity and specificity of MAPK signaling, although whether oxidative stress-induced MAPK activation is mediated by LLPS currently remains elusive.

### Transcriptional Regulation

Generally, most of the excess cellular oxidation is sensed by thiol oxidation (Baba and Bhatnagar, 2018), as exemplified by oxidation in cysteine residues of Keap1. Oxidized Keap1 molecules are prevented from binding to Nrf2, thus activating Nrf2-mediated redox regulation pathways (Suzuki and Yamamoto, 2015). An alternative Nrf2-mediated pathway, namely the p62-Keap1-Nrf2 axis, also senses cellular damages through LLPS and activates autophagy to degrade these damaged organelles (Ichimura et al., 2013; Filomeni et al., 2015; Jiang et al., 2015). p62 is a cargo receptor for the selective autophagy of ubiquitinated targets. Previous reports describe how p62 forms aggregates with ubiquitinated proteins and Keap1, a suppressor of Nrf2 function (Katsuragi et al., 2016). p62-bound Keap1 loses the ability to interact with Nrf2, which leads to Nrf2 activation. Then p62-Keap1 aggregates are degraded by selective autophagy machinery. As a result, p62 aggregation induces selective autophagy as well as Nrf2 activation in parallel (Komatsu et al., 2010). On the contrary, activated Nrf2 induces the transcription of p62, thus forming a positive feedback loop (Jain et al., 2010). Of note, recent studies show that p62 forms reversible, solid gel-like droplets, which is triggered by p62 binding to ubiquitin chains (Cloer et al., 2018; Sánchez-Martín et al., 2020; Kageyama et al., 2021). Although this p62 droplet is a target of autophagy-mediated degradation, before being degraded the p62 condensates enable reversible changes of Keap1 translocation onto the p62 condensates (Kageyama et al., 2021). This dynamic feature of p62 gels, which plays roles in the activation of both autophagy and Nrf2, provides flexibility within the cellular oxidative stress response system. Once the redox imbalance is resolved, Nrf2 needs to be quickly inactivated because prolonged Nrf2 hyperactivation leads to adverse effects such as type-1 diabetes or aging acceleration through metabolic dysregulation (Tsakiri et al., 2019). Therefore, the LLPS-mediated dynamic regulation of Nrf2 activity by Keap1 translocation onto p62 gels likely provides efficient and precise management of the stress response system.

Neighbor of BRCA1 gene 1 (NBR1), another cargo receptor for selective autophagy of ubiquitinated targets (Lamark et al., 2009), acts as a mediator of p62 droplet formation during oxidative stress via controlling p62 phosphorylation (Sánchez-Martín et al., 2020). NBR1-mediated signaling nodes through p62 droplet formation activate the Keap1-Nrf2 antioxidant pathway (NBR1-p62-Keap1-Nrf2) (Sánchez-Martín et al., 2020). NBR1 senses oxidative insult in a different way from thiol-oxidation in Keap1. The mechanism of NBR1 induction by oxidative insult is not yet fully elucidated. Nrf2 regulates NBR1 expression at least in part in the aged brain, suggesting that there is a regulatory feedback in NBR1-Nrf2 regulation (Tang et al., 2018). Conversely, it has been also shown that LLPS of p62 is suppressed by MOAP-1 (modulator-of-apoptosis-1), resulting in the activation of Nrf2 (Tan et al., 2021). MOAP-1-deficient mice exhibit enhanced tumor development in a hepatocarcinogenesis model through Nrf2 hyperactivation (Tan et al., 2021). Mechanistically, oxidative stress upregulates MOAP-1 then modulates p62 droplet formation-induced Nrf2 activation to avoid Nrf2 hyperactivation. Although the biological significance of these multiple sensors is not fully understood, the NBR1-p62-Nrf2 axis may enable precise management for redox imbalance-induced cellular damages.

**FIGURE 3:**
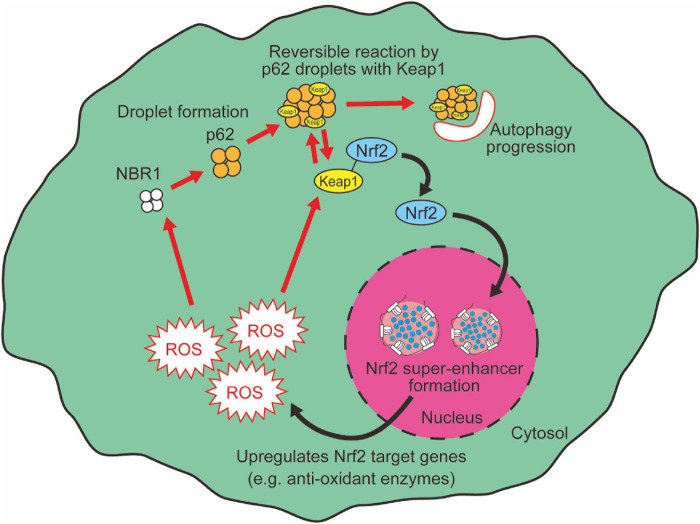
LLPS mediates Nrf2 activation.

**FIGURE 4:**
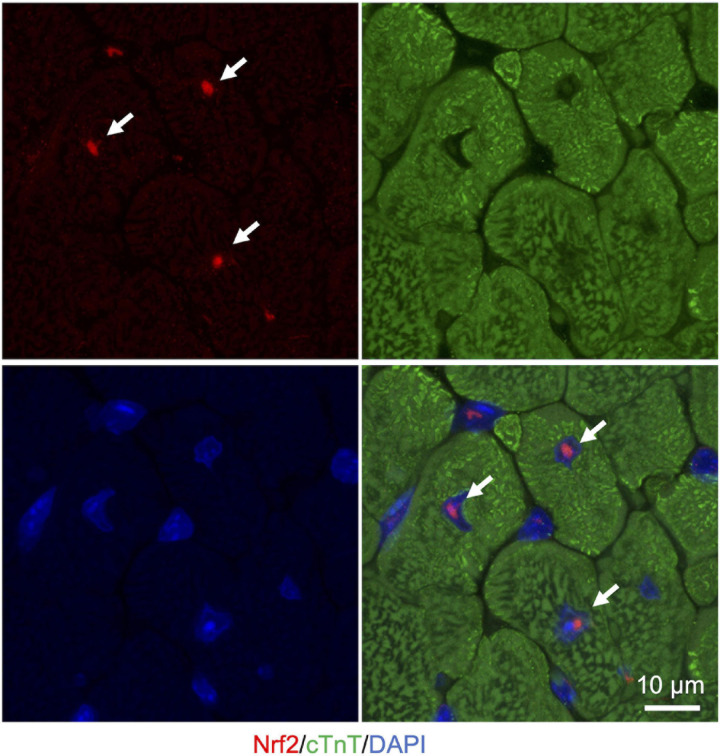
Nuclear Nrf2 condensates in the adult murine heart. Representative images of immunostaining in cryosection of heart from ICR mice (3 months of age). Nrf2 is shown in red. cTnT (cardiac troponin T), a marker for cardiomyocytes, is shown in green. Nuclei are visualized in blue with DAPI. White arrows indicate nuclear Nrf2 condensates in cardiomyocytes.

Liquid-liquid phase separation-mediated transcriptional control also contributes to the regulation of the cellular oxidative response. Clusters of enhancers called super-enhancers contain a larger number of transcription factors than a typical single enhancer and exhibit properties of liquid-like condensates (Sabari et al., 2018). A recent study reported that Nrf2 undergoes phase separation to form nuclear condensates with a mediator complex at super-enhancers (Lu et al., 2020). It is interesting to note here that several small-molecule cancer therapeutics show a trend to concentrate in phase-separated super-enhancers (Klein et al., 2020). Given that Nrf2 activity is known to define cancer malignancy (Rojo de la Vega et al., 2018), Nrf2 super-enhancer formation may either contribute to drug resistance or determine sensitivity against chemotherapy in certain types of cancer cells. Therefore, increasing evidence shows that LLPS plays a pivotal role in the regulation of the Keap1-Nrf2 antioxidant pathway through both p62-mediated pathway switching and Nrf2-mediated transcriptional regulation (Figure 3). Intriguingly, we also observed that large nuclear condensates of Nrf2 formed only a few foci per nucleus in adult murine cardiomyocytes (Figure 4). Since cardiomyocytes are known to be highly metabolically active and thus are continuously exposed to oxidative stress (Puente et al., 2014; Nakada et al., 2017), molecular rules underlying Nrf2 nuclear assembly there may well be distinct from those in relatively metabolically inactive tissues. Tissue- and cell-type specific composition and roles of Nrf2 condensates merits further investigation.

## LLPS-Mediated Resistance for Redox Imbalance

### Metabolic Switching

Acute hypoxia causes redox imbalance by metabolic disturbance (McGarry et al., 2018). Recent evidence supports the notion that LLPS serves as one of the key mechanisms by which cells deal with hypoxia to protect themselves from hypoxia-induced cellular damages. Acute hypoxia leads to Glycolytic body (G body) formation in yeast and also human hepatocarcinoma cells (HepG2 cells) (Jin et al., 2017). G bodies are phase-separated hydrogel-like membraneless organelles incorporating glycolytic enzymes (e.g. phosphofructokinase) (Jin et al., 2017; Fuller et al., 2020). Their presence correlates with an increase in glucose consumption and cell survival in a hypoxic condition (Jin et al., 2017). These phase-separated condensates of glycolytic enzymes enable efficient energy production under acute hypoxia and promote cell survival (Jin et al., 2017). Therefore, G body formation possibly contributes to metabolic switching from mitochondrial energy synthesis to glycolysis. This metabolic switching not only enables cells to produce ATP efficiently under oxygen-deprived conditions, but also closely relates to cellular redox state as acute hypoxia induces ROS production at the Q_*o*_ site of the mitochondrial complex III (Bell et al., 2007). Of note, arsenite-induced oxidative stress did not trigger G body formation (Jin et al., 2017), indicating that oxidative stress is not a direct cause for this metabolic switching. Mechanistically, AMPK signaling, a sensor of cellular energy levels, has been identified as a regulator of G body formation in yeasts (Jin et al., 2017). AMPK increases glycolysis as a part of an adaptive response to oxidative stress (Wu and Wei, 2012). G body formation possibly contributes to the prevention of mitochondrial metabolic disturbance-associated redox imbalance through glycolysis-oriented metabolic switching. Whether hypoxia triggers association or dissociation of molecular condensates other than G bodies, and if so, what their physiological significances are, are interesting directions for future research.

### Translational Remodeling

When cells are suffering from redox imbalance, they stall homeostatic mRNA translation. Following stalled translation initiation under various stress conditions, stress-responsive phase-separated membraneless organelles called stress granules (SGs) are formed (N Kedersha and Anderson, 2002). SGs are induced by the phosphorylation of eukaryotic initiation factor (eIF) 2α and/or eIF4s (N Kedersha and Anderson, 2002; Panas et al., 2016), and physically segregate mRNA and translation machinery to prevent untranslated RNA from oxidative damage (Kedersha et al., 2013). The components of SGs are not limited to RNA or the machinery of RNA translation but include several regulatory proteins of translation. Mammalian target of rapamycin complex1 (mTORC1), which regulates RNA translation (Thoreen et al., 2012), is known to be divided into its component proteins mTOR and raptor, and recruited into SGs under the stress condition (Heberle et al., 2015). This segregation of mTORC1 components also contributes to a transient arrest of RNA translation in the stress condition (Heberle et al., 2015). On the other hand, some types of mRNAs escape from being incorporated into SGs and undergo translation at the ER to respond to stress-induced cellular damage (Unworth et al., 2010). Also, mTORC1 activity is not completely inhibited in the stress condition. The expression of several stress response proteins (e.g., ATF4 and Hsp70) are upregulated by mTORC1 under the stress condition (Heberle et al., 2015).

In the oxidative stress condition, the formation of stress granules protects cells from apoptosis in a distinct manner. A key factor by which SGs inhibit apoptosis is the signaling scaffold protein RACK1. In the oxidative stress condition, RACK1 is sequestered into SGs, which inhibits the activation of MAPK signaling, and as a result the apoptosis signals are suppressed (Arimoto et al., 2008). In contrast, SG formation is not induced by direct DNA damage with X-ray irradiation or genotoxic drugs (e.g. etoposide); instead, in these cases p38 and JNK MAPK pathways induce apoptosis (Arimoto et al., 2008).

Another stress-induced membraneless organelle, the processing bodies (PBs) also provide sites for sequestration of untranslated RNAs (Jain and Parker, 2013). PBs contain the 5′–3′ mRNA decay machinery, and thus some population of mRNAs within PBs are degraded, whereas mRNAs within SGs are not degraded (Sheth and Parker, 2003; Cougot et al., 2004). Also, contrary to SGs, PBs are observed in normal physiological states in addition to oxidative stress condition (Kedersha et al., 2005). Moreover, DNA replication stress increases the number of PB and PB-involved stress resistance (Tkach et al., 2012; Loll-Krippleber and Brown, 2017). The transcription factor YOX1 normally downregulates genes that confer resistance to DNA replication stress (Loll-Krippleber and Brown, 2017). Once DNA replication stress is induced by hydroxyurea, stress-induced PBs sequester YOX1 mRNAs and degrade them, which upregulates YOX1-target genes to activate stress-response gene programs (Loll-Krippleber and Brown, 2017).

Although SGs and PBs are distinctly phase-separated, these two cytoplasmic bodies interact with each other to exert a proper response to cellular stresses. Upon exposure of cells to multiple stressors, firstly polysomes are disassembled (Anderson and Kedersha, 2008; Ross Buchan, 2014; Jayabalan et al., 2016), and then the mRNAs are sorted at SGs, and also part of them are incorporated in PBs to be degraded (Nancy Kedersha et al., 2005). Indeed, in some contexts, PBs and SGs share some protein and mRNA components, although the components of PBs and SGs vary depending on the types of stressors, cells, and organisms (Nancy Kedersha et al., 2005). Moreover, recent studies suggest multiple and fine-tuned regulations of cellular stress responses mediated by LLPS. Canonically, SGs uniformly inhibit translation of segregated mRNA (Anderson and Kedersha, 2008; Buchan and Parker, 2009), as exemplified in cancer cells where SGs inhibit HIF-1 signaling by partial sequestration of the HIF-1-regulated transcripts (Moeller et al., 2004). However, a recent study with single-molecule imaging under arsenite-induced oxidative stress demonstrated that some mRNAs localized at SGs are compatible with translation (Mateju et al., 2020). As shown by this latest update, the physical properties, physiological roles, and molecular functions of SGs are not yet completely understood.

### DNA Damage Response

As mentioned in the introduction, excessive ROS cause oxidative DNA damages which lead to genomic instability and mutations (Lonkar and Dedon, 2011; Mangerich et al., 2012; Cadet and Davies, 2017). To prevent tumorigenic mutation, cells must precisely recognize the sites of DNA damage and appropriately respond to them (Poetsch, 2020). Ataxia-telangiectasia mutated (ATM) is one of the upstream kinases that initiates the DNA damage response (DDR) signaling cascade by phosphorylation of histone variant H2AX (resultant phosphorylated H2AX is usually called γH2AX), as well as many other phosphorylation substrates (Goodarzi et al., 2008; Shiloh and Ziv, 2013; Blackford and Jackson, 2017). γH2AX is centered at damaged DNA loci and forms a large complex with various DDR-associated factors to recruit p53-binding protein 1 (53BP1) (Panier and Boulton, 2014). 53BP1 foci at damaged chromatin serve as a scaffold for the downstream DDR effector assembly, and protect DNA lesions against excessive nucleolytic digestion (Panier and Boulton, 2014; Kilic et al., 2019). Of note, Kilic et al. (2019) revealed that phase separation of 53BP1 regulates localized DNA damage recognition and repair factor assembly. As a regulatory mechanism of 53BP1 condensation, it has been proposed that damage-induced long non-coding RNAs drive molecular crowding of DDR proteins including 53BP1 into γH2AX foci (Pessina et al., 2019). Phase separated liquid droplets of 53BP1 recruit a tumor suppressor protein p53, and resultant enrichment of p53 activates the DDR pathway (Kilic et al., 2019).

Another type of DDR-related liquid droplets utilizes cytoskeletal networks to enhance clustering of damaged DNA sites (Chung et al., 2015; Oshidari et al., 2018). Intriguingly, the damaged DNA repair is dynamically regulated by motor protein complexes and intranuclear filaments. In addition, DNA damage-inducible intranuclear microtubule filaments (DIMs) directionally mobilize damaged DNA to nuclear pore complexes and promote DNA repair (Chung et al., 2015; Oshidari et al., 2018). Oshidari et al., showed DIMs drive the fusion of the liquid droplets of DNA repair protein Rad52, mediating the clustering of damaged DNA sites to promote the function of the DNA repair droplets (Oshidari et al., 2020).

Levone et al. (2020) reported that LLPS of the RNA-binding protein FUS (Fused in Sarcoma) regulates the initiation of DDR signaling (Levone et al., 2021). FUS-dependent LLPS is required for the formation of γH2AX and 53BP1 foci formation, and proper recruitment of DDR-associated proteins (Levone et al., 2021). Although the phase separation capacity of FUS is well-characterized (Patel et al., 2015; Murray et al., 2017), there remains room for further exploration of the phase behavior and dynamics of FUS within nuclei. Given mutations in FUS associate with ALS pathogenesis, LLPS of FUS may also contribute to the etiology of neurodegenerative diseases (Patel et al., 2015). TDP-43 also associates with degeneration of motor neurons in ALS patients (Arai et al., 2006; Neumann et al., 2006). Similar to FUS, TDP-43 forms liquid droplets containing gel-like cores in the cytosol (Maharana et al., 2018; Sun and Chakrabartty, 2017). Mitra et al. (2019) reported that nuclear TDP-43 participates in the DDR and mislocalization of TDP-43 from nucleus to cytoplasm causes neurodegeneration with persistent genome damage. The contribution of their downstream cytosolic aggregates formation to ALS pathogenesis is not fully understood.

### Lipid Droplets

**FIGURE 5:**
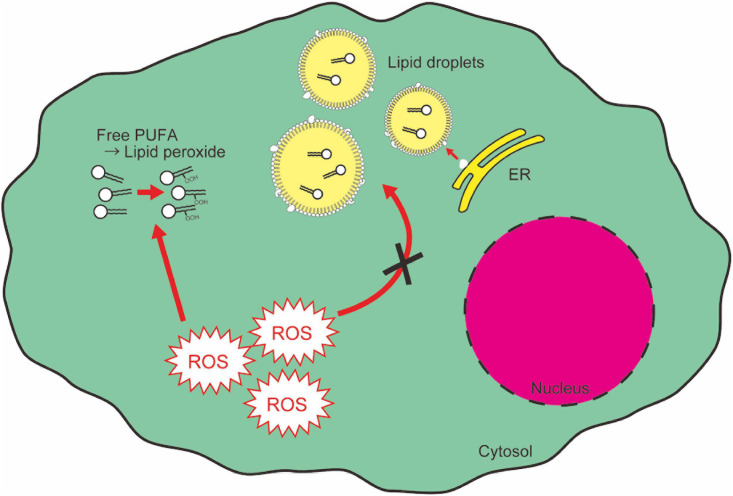
Lipid droplets segregate and protect PUFAs from cytotoxic peroxidation. Free PUFAs are susceptible to oxidation and converted into lipid peroxides which cause cytotoxic insult as a second messenger of ROS signaling. ER, endoplasmic reticulum.

Fatty acids and their derivatives are critical for cellular oxidative response not only because lipid peroxidation is one of the main causes of cellular oxidative damage, but also because lipids provide protective machinery against cellular oxidative damage. PUFAs are an important source of functional cytosolic membrane and mitochondrial energy production, and they are susceptible to auto-oxidation (Farmer et al., 1943; Gardner, 1989). The end products of lipid peroxidation [e.g., 4-hydroxynonenal (HNE)] work as a “second messenger” of oxidative stress because of their greater diffusability than free radicals (Barrera, 2012). This property gives lipid peroxides another toxicity to cells. On the other hand, several reports show the role of intracellular lipid droplets in protecting cells from cytotoxicity of lipid peroxidation. Lipid droplets have an electrically neutral lipid core consisting of triglycerides and sterol esters, surrounded by a charged phospholipid monolayer (Martin and Parton, 2006; Thiele and Spandl, 2008; Walther and Farese, 2012). Lipid droplets can incorporate PUFAs within their lipid core, which enables compartmentalization and prevention of PUFAs from oxidation, and thereby from causing cytotoxicity (Figure 5; Jarc and Petan, 2019). This lipid droplet-mediated protection of cells from oxidative stress has been observed in various biological contexts. In the neural stem cell niche in *Drosophila*, oxidative stress increases the formation of lipid droplets which accumulate cytoplasmic membranes and protect membranes from peroxidation reactions (Bailey et al., 2015). In addition, in tumor cells, hypoxia causes lipid droplet accumulation, which contributes to cell survival after hypoxia-reoxygenation (Bensaad et al., 2014). In the hypoxic condition, although *de novo* fatty acid synthesis is repressed, there is an enhancement in FABP3/7-dependent fatty acid uptake, which causes lipid droplet accumulation (Bensaad et al., 2014). FABP7-induced lipid uptake and lipid droplet formation also protects astrocytes from hypoxic damage (Islam et al., 2019). Recent reports indicate, however, that over-accumulation of lipid droplets causes neurotoxicity. In aged mouse and human brains, microglia accumulating lipid droplets are observed (Marschallinger et al., 2020). These microglia show impaired phagocytosis activity and produce high levels of ROS, resulting in age-related neurodegeneration (Marschallinger et al., 2020). Therefore, while lipid droplets confer resistance against oxidative reagents, which are required for cell survival in stress conditions, chronic accumulation of lipid droplets also leads to detrimental side effects.

Lipid droplet is a canonical example of LLPS (Thiam and Forêt, 2016; Thiam, 2020). Lipid droplet biogenesis originates in the endoplasmic reticulum (ER) (Brasaemle and Wolins, 2012; Pol et al., 2014). Under nutrient-rich conditions, excess carbohydrates are converted to triglycerides and sterol esters. However, the mechanistic basis of how triglycerides and sterol esters form intracellular lipid droplets remains largely unknown (Thiam and Forêt, 2016). Very recently, Zoni et al. (2020) reported that lipid droplet biogenesis is driven by LLPS. The physiological properties of specific lipid composition of the ER enable the packaging of neutral lipids into lipid droplets without being accumulated in the ER membrane (Zoni et al., 2020). When cells are starved, lipid droplet degradation by neutral lipases, namely lipolysis, progresses to utilize fatty acids reserved in lipid droplets as a source of mitochondrial energy production by fatty acid oxidation (Lass et al., 2011). Lipolysis is regulated by mTORC2 (Kumar et al., 2010; Lamming and Sabatini, 2013): suppression of mTORC2 activity leads to higher basal levels of lipolysis (Kumar et al., 2010). Lipid droplet degradation also progresses in an autophagy-associated manner, called lipophagy, a selective form of macroautophagy (Cingolani and Czaja, 2016). Altogether, lipid droplets are dynamic lipid compartments that enable flexible use of unstable cellular lipids.

## Pathology-Associated Phase Separation

As described in the section “Lipid Droplet,” chronic accumulation of LLPS-mediated condensates can be detrimental to cellular survival (Marschallinger et al., 2020). Another example in which phase-separated droplets are harmful to cells is amyloid fiber formation. Firstly, the first step of amyloid aggregation is mediated by LLPS, and secondly, liquid-solid phase separation (LSPS) of amyloid fibers, such as α-Synuclein, causes redox imbalance, impairing healthy organelles function (Ray et al., 2020).

Oxidative stress has been considered to be one of the causes of amyloid-induced pathologies [e.g., Alzheimer’s disease, Parkinson’s disease, and amyotrophic lateral sclerosis (ALS)]. In Amyloid β (Aβ)-induced neurotoxicity, Aβ impairs mitochondrial redox activity and increases the generation of ROS (Behl et al., 1994; Hensley et al., 1994; Shearman et al., 1994). Mitochondria-targeted α-Synuclein overexpression increases mitochondrial ROS production, reduces ATP levels, and causes disruption of dendritic neural network structure (Ganjam et al., 2019). In addition, Musgrove et al. reported that over-expression of α-Synuclein in the dorsal motor nucleus of the vagus nerve (DMnX), a primary site of pathological α-Synuclein deposition, caused elevated ROS production (Musgrove et al., 2019). In this context, exposure of these nerve cells to the ROS-generating agent paraquat leads to enhanced production of both ROS and an oxidatively modified form of α-Synuclein. Notably, enhanced ROS production affected neuron-to-neuron protein transfer which spreads α-Synuclein from the DMnX toward other brain regions (Musgrove et al., 2019). Considering the protective or stress-resistant features of previously described LLPS-mediated membraneless organelles, one of the biological roles of amyloids is considered to be the protection of cells from stress (Kroschwald and Alberti, 2017; Franzmann et al., 2018). However, several types of amyloids are susceptible to oxidation, and oxidized amyloid fibers then cause neurotoxicity by oxidation chain reaction (Musgrove et al., 2019). To prevent this undesirable exacerbation of Aβ toxicity in neuronal networks, microglia would be an attractive therapeutic target. Genetic intervention to suppress microglial membrane ROS production significantly reduced α-Synuclein neurotoxicity (Musgrove et al., 2019). LC3-associated endocytosis in microglia serves as a clearance mechanism for Aβ aggregates (Heckmann et al., 2019). Microglial redox balance is susceptible to being disrupted under several pathological conditions, as indicated by ROS-associated lipid droplet accumulation in dysfunctional microglia in aged brains (Marschallinger et al., 2020). Therefore, maintenance of microglial redox balance is a potential therapeutic strategy to prevent amyloid-induced pathogenesis.

The degradation of several types of droplets (e.g., stress granules, lipid droplets, and Pbp1 droplets) is regulated by mTOR-mediated autophagy machinery (Kumar et al., 2010; Buchan et al., 2013; Anding and Baehrecke, 2017; Kato et al., 2019, 2021), indicating autophagy plays critical roles in proper droplet disassembly. Rapamycin-induced autophagy contributes to the reduction of Aβ level (Caccamo et al., 2010). However, starvation-induced autophagy is not sufficient for Aβ clearance (Chen et al., 2015). What makes the difference between these two autophagy pathways in amyloid degradation is controversial (Caccamo et al., 2010; Chen et al., 2015). Finding missing pieces of factors for clearance of LLPS- and LSPS-derived cellular debris is anticipated for future clinical application.

## Concluding Remarks

Increasing evidence indicates that LLPS closely associates with cellular redox maintenance. However, *in vivo* evidence, especially in mammals, showing direct roles of LLPS in redox maintenance is still limited. This is mainly due to the shortage of applicable methods to test the biological roles of LLPS without changing factors that are intertwined with LLPS, including protein conformation, cellular physiology such as pH or ionic strength, or gene/protein expression. Remarkably, Quiroz et al. (2020) provided *in vivo* phase separation sensors to show how LLPS contributes to skin barrier formation. To evaluate phase separation dynamics *in vivo*, they combined synthetic phase separation sensors that minimally interfere with phase separation behavior of target molecules with an *in utero* lentiviral delivery system, and directly assessed *in vivo* phase separation with live imaging (Quiroz et al., 2020). Although currently live imaging of LLPS in animals including mice is restricted to external tissues, their approaches can be applicable for further evaluation of *in vivo* phase separation events. Researchers have been using novel approaches to better understand biological phase separation. Mateju et al. (2020) revealed continued translation of mRNA within stress granules using SunTag-based single-molecule imaging. SunTag-based imaging enables visualization of individual reporter mRNAs (Pichon et al., 2016; Wang et al., 2016; B. Wu et al., 2016; Yan et al., 2016). Combinatorial approaches of this single-molecule imaging with *in vivo* live imaging may enable more complete and detailed measurement and depiction of phase separation in biological phenomena such as embryonic development, disease states, and biological evolution.

While the relevance of LLPS in multiple aspects of redox biology is increasingly becoming evident, a major stumbling block has been the challenge of directly assessing the role of LLPS *in vivo*. To directly investigate *in vivo* roles of a biological phase separation event, one must artificially and accurately manipulate LLPS at will. For this purpose, several optogenetic or chemical manipulation tools have been developed (Shin et al., 2017; Dine et al., 2018; Nakamura et al., 2018). Cryptochrome-2 (Cry2) is a photoreceptive protein in *Arabidopsis thaliana* (Bugaj et al., 2013; Taslimi et al., 2014), and is used as an optogenetic tool because Cry2 forms rapid and reversible protein oligomerization in response to blue light (Bugaj et al., 2013; Taslimi et al., 2014). Shin et al. (2018) developed a tool called “optoDroplet,” which enables light-inducible droplet formation by fusing an intrinsically disordered region of a protein with Cry2. This system provides a way to directly control droplet formation with blue light (Shin et al., 2018). Conversely, a light-dissociable optogenetic tool called “PixELLs” was also developed (Dine et al., 2018) by utilizing the association of cyanobacterial proteins PixD and PixE into large multi-subunit complexes in dark conditions (Masuda et al., 2004; Yuan and Bauer, 2008). Dine et al. (2018) fused PixD and PixE with intrinsically disordered regions of proteins so that the liquid droplets are dissociated upon light exposure, enabling long-term chase of liquid droplets without light toxicity. In addition, Nakamura et al. (2018) introduced a chemically inducible LLPS manipulation, which is called “iPOLYMER.” In this system, FK506-binding protein (FKBP) and FKBP12-rapamycin-binding domain (FRB) dimerize in the presence of rapamycin (Derose et al., 2013; Nakamura et al., 2018). When multiple FKBPs are tandemly repeated with flexible linkers, and mixed with similar polymers consisting of tandemly repeated FRBs, these iPOLYMER products form a hydrogel-like phase-separated structure upon treatment with rapamycin (Nakamura et al., 2018). Chemically induced association and dissociation of biomolecules would be particularly important for manipulating LLPS in multicellular and non-transparent organisms, including mice or humans. Moreover, from a translational standpoint, direct manipulation of LLPS *in vivo* in mammals possibly provides mechanistic insights into the process of pathological amyloid formation, as well as novel therapeutic approaches for LLPS-associated pathology (Wheeler, 2020). However, the roles of biological phase separation in oxidative stress response are complex: it both causes oxidative stress and protects cells from oxidative stress. Building a better understanding of the roles of LLPS in redox regulation may lead to the invention of new tools for preventing or curing oxidative stress-related diseases.

## Author Contributions

YS and WK designed and conducted research, and contributed to manuscript preparation. Both authors contributed to the article and approved the submitted version.

## Conflict of Interest

The authors declare that the research was conducted in the absence of any commercial or financial relationships that could be construed as a potential conflict of interest.
